# Supplementary material for: Structure and dynamics of the drug-bound bacterial transporter EmrE in lipid bilayers
Source: Nat Commun. 2021 Jan 8;12:172. doi: 10.1038/s41467-020-20468-7 (PMC7794478; doi:10.1038/s41467-020-20468-7)
Supplement: Supplementary file 1 — Supplementary Information [file 41467_2020_20468_MOESM1_ESM.pdf]

Supplementary Information

Structure and Dynamics of the Drug-Bound Bacterial Transporter EmrE in Lipid Bilayers

Alexander A. Shcherbakov [1], Grant Hisao [2], Venkata S. Mandala [1], Nathan E. Thomas [2], Mohammad Soltani [3], E. A. Salter [3], James H. Davis Jr.[3], Katherine A. Henzler-Wildman [2]*, and Mei Hong [1]*

[1] Department of Chemistry, Massachusetts Institute of Technology, 170 Albany Street, Cambridge, MA 02139

[2] Department of Biochemistry, University of Wisconsin at Madison, Madison, WI 53706

[3] Department of Chemistry, University of South Alabama, Mobile, AL 36688

This PDF file includes Supplementary Figures 1 to 10

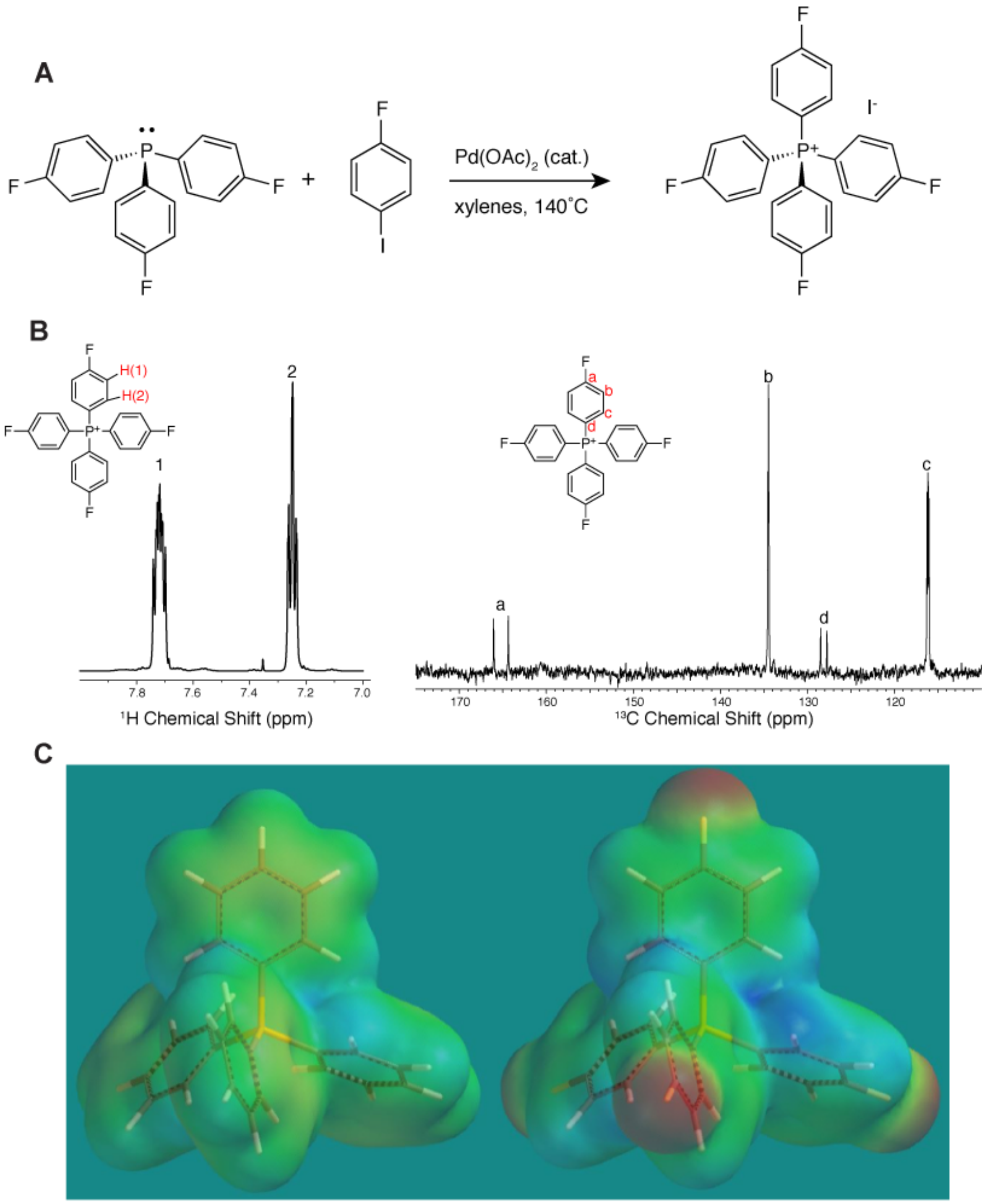

Supplementary Figure 1. F_4-TPP^+ synthesis. (**A**) Synthetic mechanism of tetra(4-fluorophenyl) phosphonium iodide. (**B**) 1D ^1H (left) and ^{13}C (right) solution NMR spectra of F_4-TPP^+. Substrate was dissolved in $CDCl_3$ and data were collected on a 600 MHz spectrometer. Chemical shift assignments are indicated. (**C**) Electrostatic potential surfaces calculated for TPP^+ (left) and F_4-TPP^+ (right) exhibit differences in electron density distribution. The electronegativity of the fluorine atoms in F_4-TPP^+ make the phosphorus center more electropositive, which is responsible for the differences in affinity and chemical shift upon binding to EmrE. Color ranges correspond to potential energies ranging from 145.1 kJ/mol (red) to 421.9 (blue) kJ/mol. The max and min energy for TPP^+ is 381.4 kJ/mol and 220.5 kJ/mol. The max and min energy for F_4-TPP^+ is 421.9 kJ/mol and 145.1 kJ/mol.

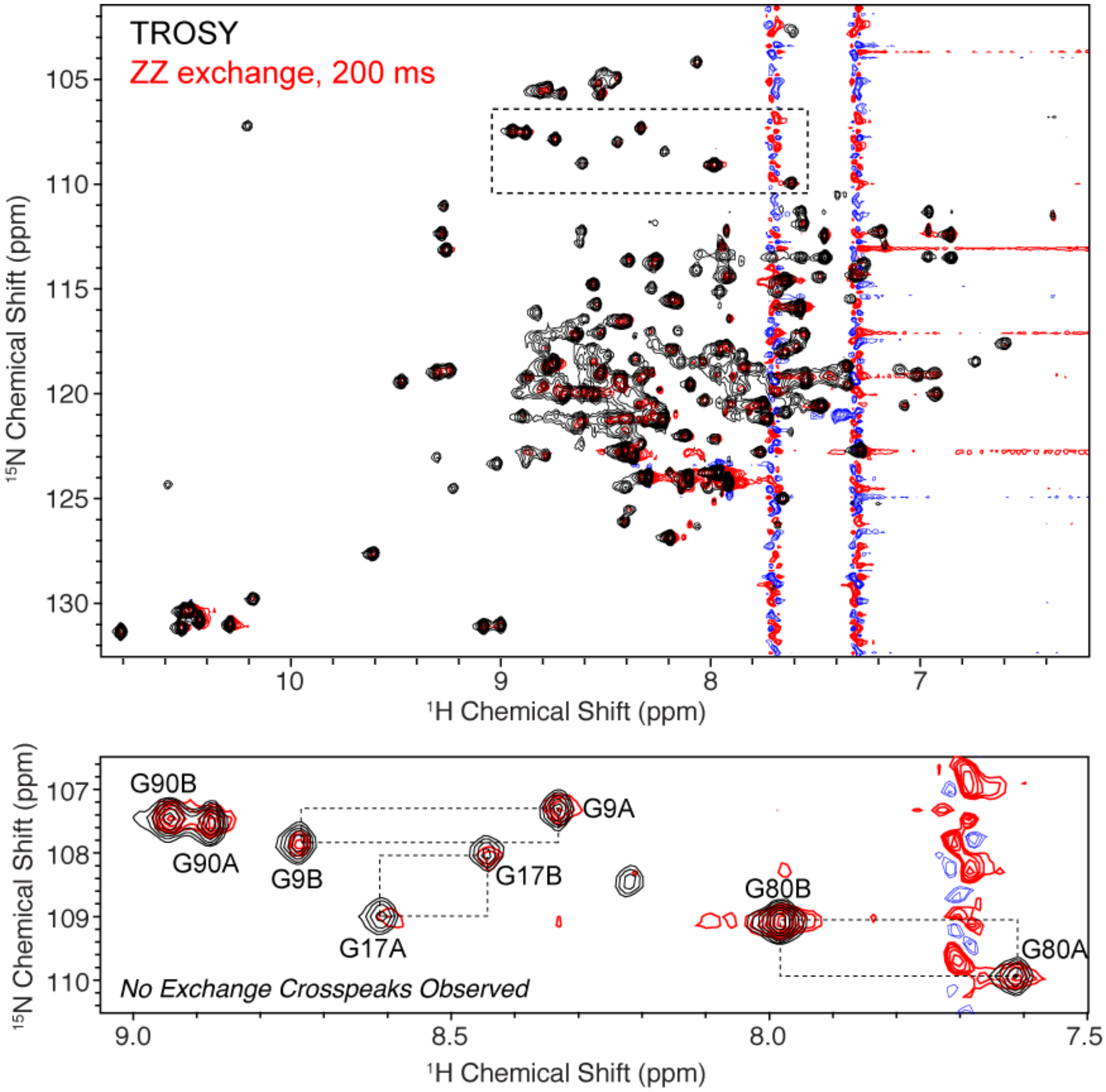

Supplementary Figure 2. Solution NMR 2D ^{1}H-^{15}N ZZ exchange spectrum with 200 ms mixing (red) of ^{2}H, ^{15}N-labeled S64V-EmrE, overlaid with a control TROSY spectrum (black). The protein was reconstituted in DMPC/DHPC bicelles at a 75 : 1 lipid : monomer ratio and the spectra were measured at 45 °C, pH 5.8. No exchange peaks were observed, as highlighted in the resolved glycine region of the spectrum. Exchange peaks are quantifiable for S64V-EmrE bound to TPP^{+} under matched conditions [1], indicating that the protein conformational exchange in the presence of bound F_4-TPP^{+} is slower than 0.5 s^{-1}.

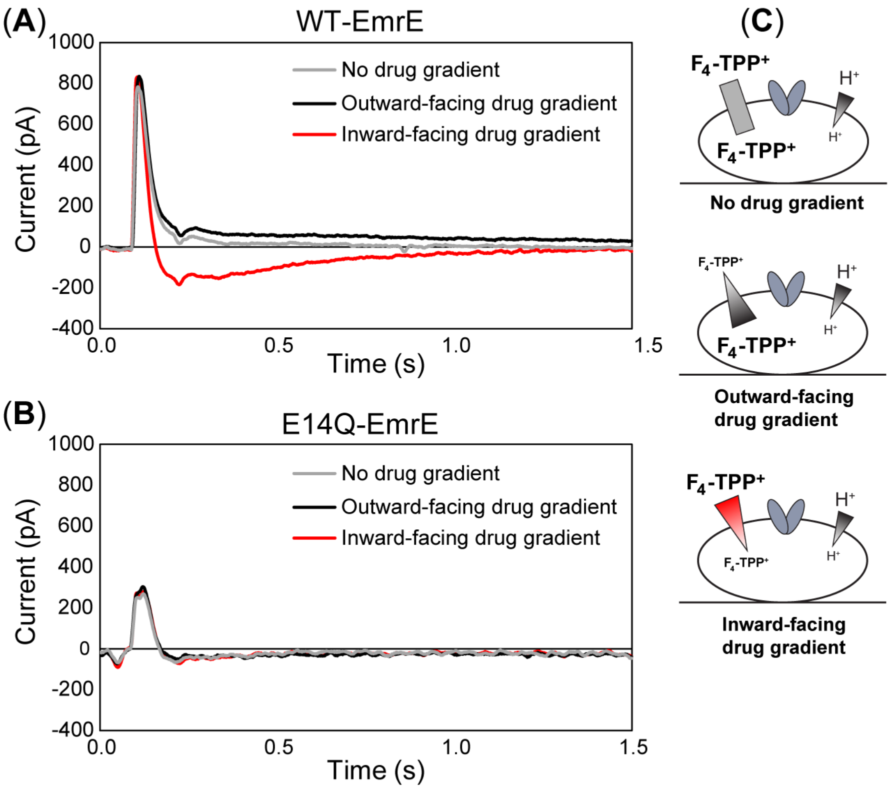

Supplementary Figure 3. Representative solid-supported membrane-based electrophysiology data of F_4-TPP^+ transport by EmrE. **(A)** Current traces for WT-EmrE. **(B)** Current traces for the transport-dead mutant E14Q-EmrE. (**C**) Schematic of the TPP^+ gradients in the different experiments, reproduced from Figure 1 for clarity. At time 0, perfusion of an external buffer set a 2-fold inward-facing H^+ gradient and a 16-fold F_4-TPP^+ gradient (or no gradient) facing the indicated direction. The current trace is at least biphasic for all conditions, with an initial fast positive current (indicating positive charges moving into the liposomes) followed by a slower process with a directionality determined by the gradient conditions. As there is a brief positive spike in the transport-dead E14Q samples, it is likely that some fraction of the positive spike in the WT samples is due to solution exchange artifacts, but this alone cannot account for the much larger spike in the WT samples. In assigning the slow and fast components, we consider several points: 1) It is not uncommon to observe a fast initial signal that represents a non-turnover process, such as substrate binding, and a slower component corresponding to transport [2]. Substrate binding is independent of the transmembrane gradients, and thus should not reverse, which is observed for the fast component. Thus, the initial fast component may represent substrate binding. 2) Substrate-on rates are fast and alternating access of proton-bound EmrE is about an order of magnitude faster than drug-bound EmrE [3]. Furthermore, both substrate-on and alternating access of proton-bound EmrE are essentially independent of drug gradient, and thus should be the same under all conditions. The initial spike is nearly identical under all gradients and it is only the falling edge that varies as the magnitude of the slow component contributes to the signal. Thus, the initial fast component may also represent turnover of proton-bound EmrE, if such turnover is triggered by binding of F_4-TPP^+. 3) The NMR data shows that the alternating-access rate of EmrE bound to F_4-TPP^+ is $\leq 0.5\ s^{-1}$, and this constrains the rate of net turnover as well. The slow process occurs on the timescale of a second, consistent with this limiting rate for net transport of F_4-TPP^+. Ultimately, while there is not enough data here to definitively determine what processes are represented in the different current phases, the more important parameter is the total transported charge (integrated current), which is shown in Figure 1. The amount and direction of transport is a function of the thermodynamics of the system, and the reversal of the direction of transported charge (note that the slow current switches from positive to negative)

when both gradients are inwardly directed unambiguously demonstrates that a sufficiently large drug gradient can reverse proton-driven transport [4], thus F_4-TPP^+ is a proton-coupled antiported-substrate of EmrE.

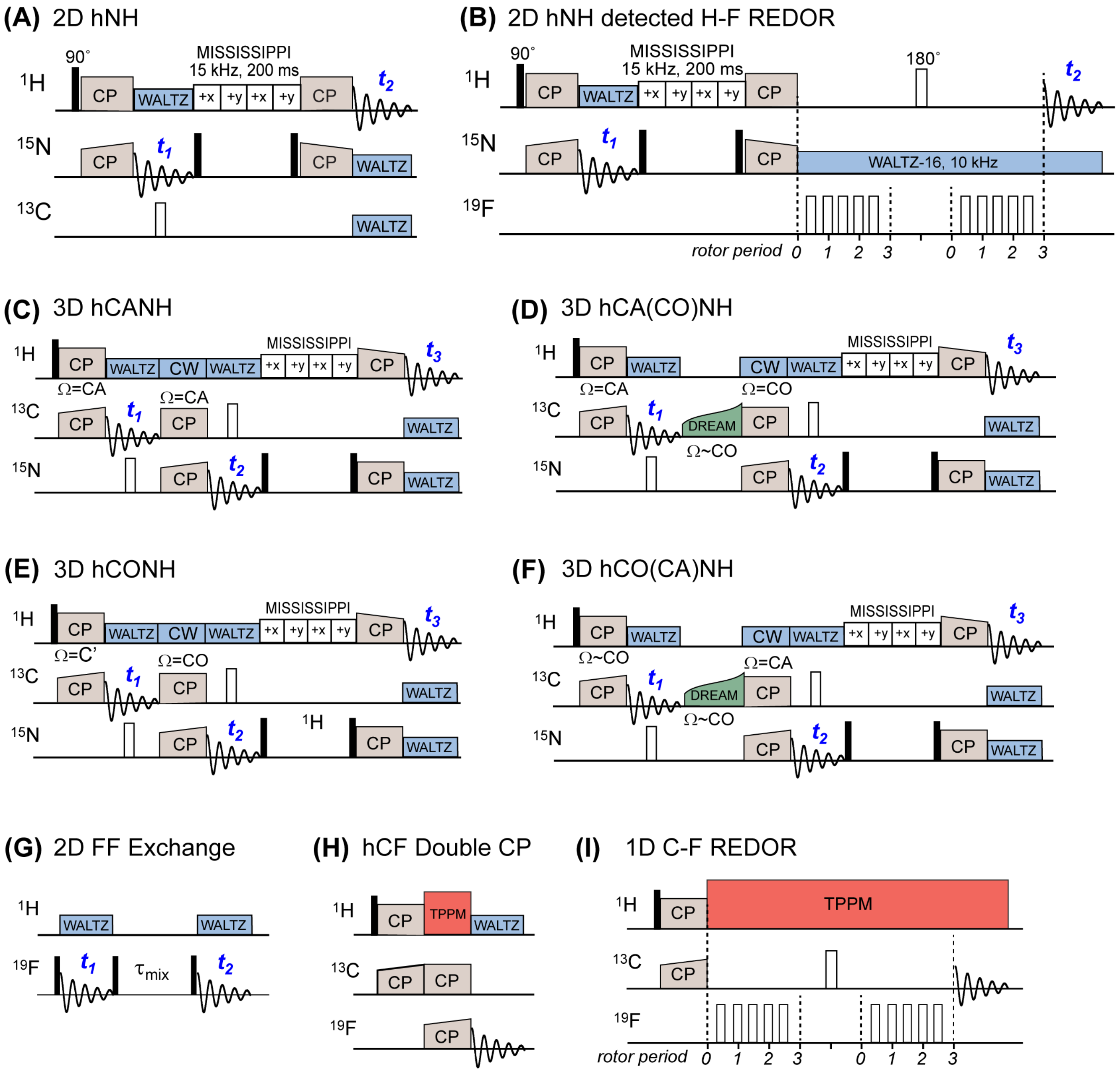

Supplementary Figure 4. Pulse sequences for ^{1}H-detected and ^{19}F-based MAS NMR experiments. (**A**) 2D hNH HSQC experiment. (**B**) 2D hNH-resolved ^{1}H-^{19}F REDOR experiment. (**C-F**) 3D ^{1}H-detected correlation experiments for resonance assignment. (**C**) Intra-residue 3D hCANH correlation experiment. (**D**) Inter-residue hCA(CO)NH correlation experiment. (E) Inter-residue hCONH correlation experiment. (**F**) Intra-residue hCO(CA)NH correlation experiment. (**G**) 2D ^{19}F-^{19}F spin exchange experiment. (**H**) 1D ^{13}C-^{19}F double-quantum CP experiment. (**I**) 1D ^{13}C-^{19}F REDOR experiment.

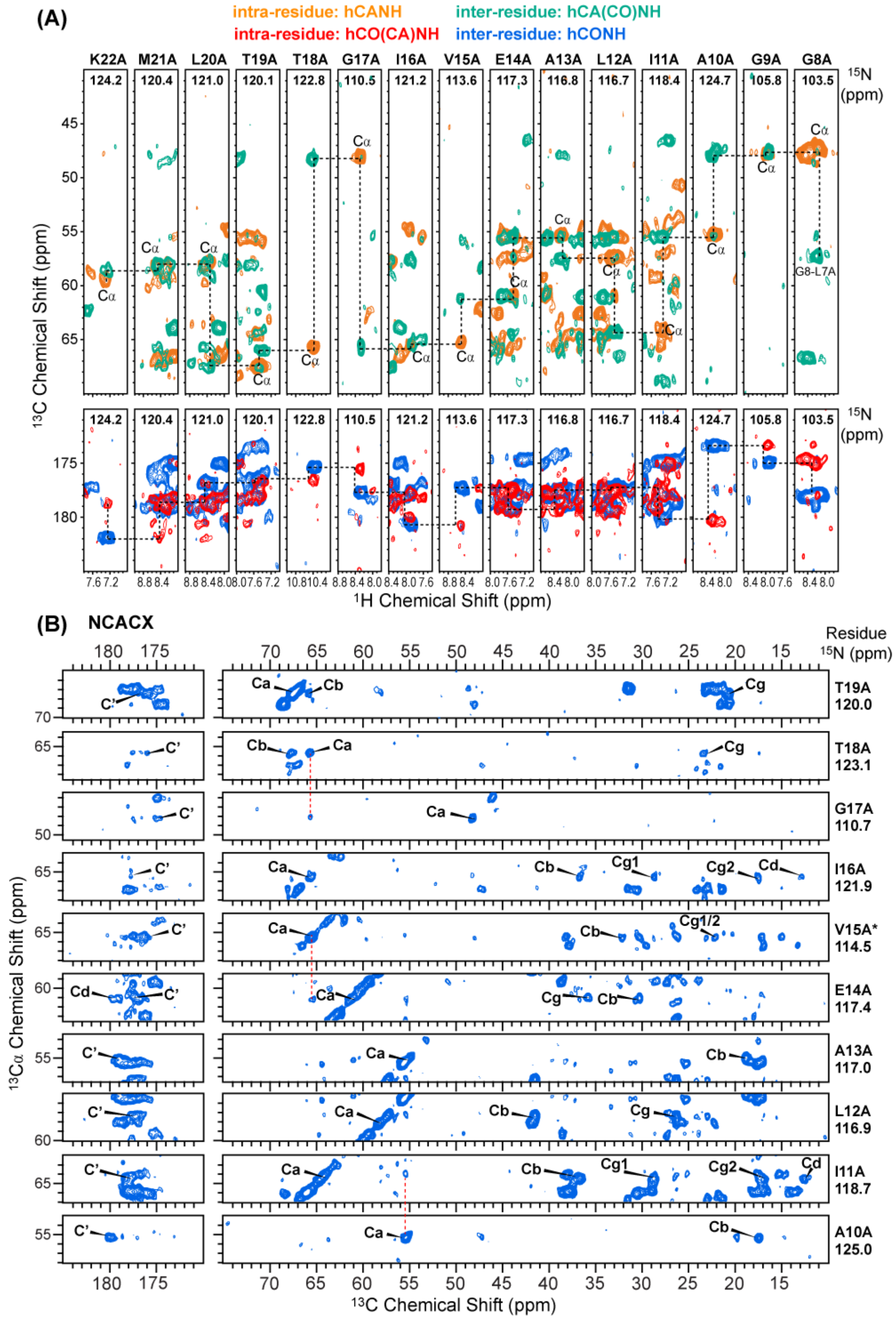

Supplementary Figure 5. Additional 3D spectral strips to illustrate resonance assignment of F_4-TPP^+ bound S64V-EmrE in DMPC bilayers. **(A)** ^{1}H-detected spectra for residues G8 to K22 in monomer A. Shown at the top are hCANH and hCA(CO)NH spectra for assigning the Cα chemical shifts, and at the bottom are hCO(CA)NH and hCONH spectra for assigning the CO chemical shifts. The spectra were measured under 55 kHz MAS on CDN-labeled protein. **(B)** Representative strips of the 3D NCACX spectrum for assigning sidechain ^{13}C chemical shifts. The spectrum was measured under 14 kHz MAS using CN-labeled S64V-EmrE.

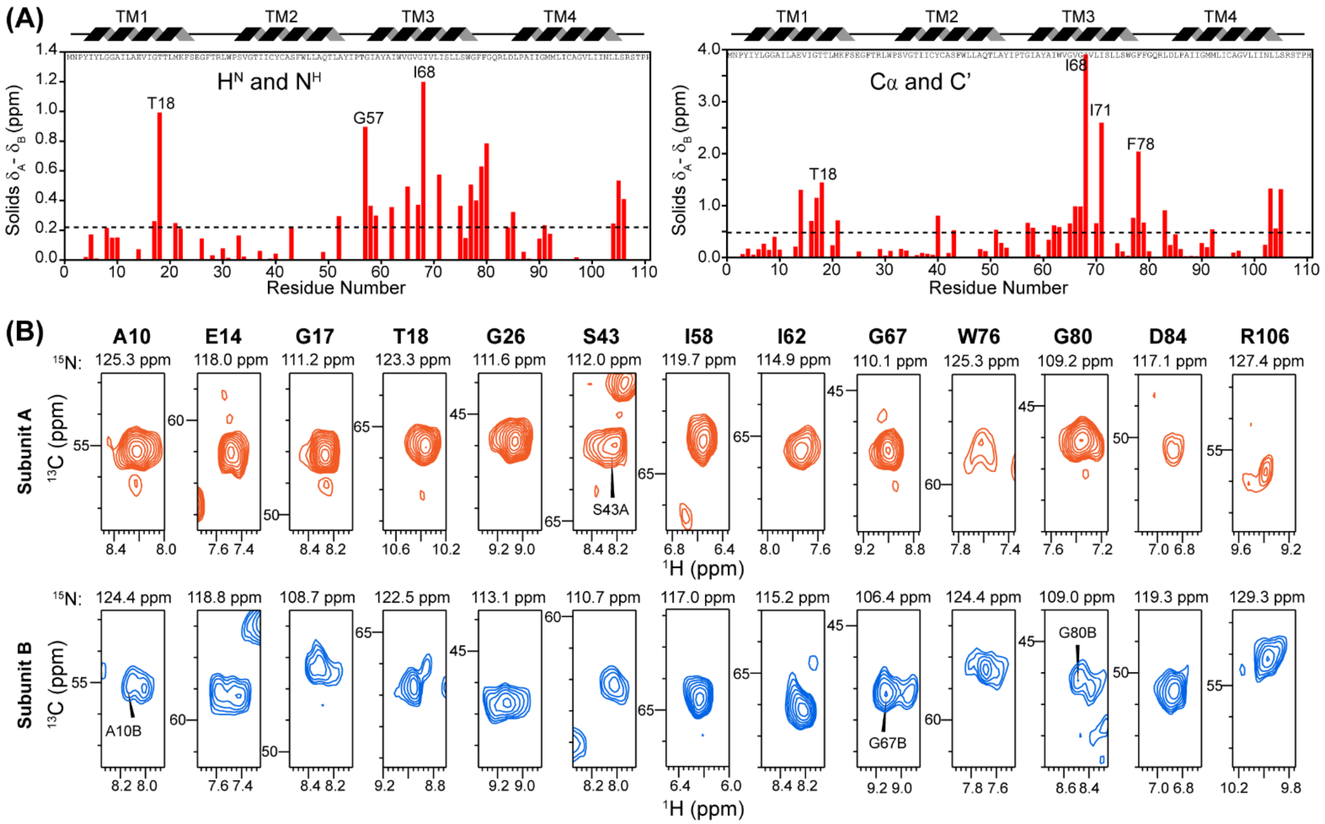

Supplementary Figure 6. Asymmetric conformation and dynamics of subunits A and B of the S64V-EmrE dimer in DMPC bilayers obtained from 3D MAS NMR spectra. (**A**) Structural asymmetry between subunits A and B of F_4-TPP$^+$ bound S64V-EmrE seen from the chemical shifts. Composite H^N and ^{15}N chemical shift differences (left) and composite Cα and CO chemical shift differences (right) are plotted. Dashed lines indicate the linewidth-based estimate of the significance levels for chemical shift perturbation, which are 0.22 ppm for the amide and 0.5 ppm for ^{13}C. **(B)** Peak intensity differences between subunit A (orange) and subunit B (blue) residues in the dimeric protein. The A peaks are stronger than the B peaks in the N-terminal half of the protein, become more comparable in the middle of TM3 (around residue I62), then become weaker than the subunit B peaks for residues near the C-terminus. Thus the two monomers have opposite dynamic gradients.

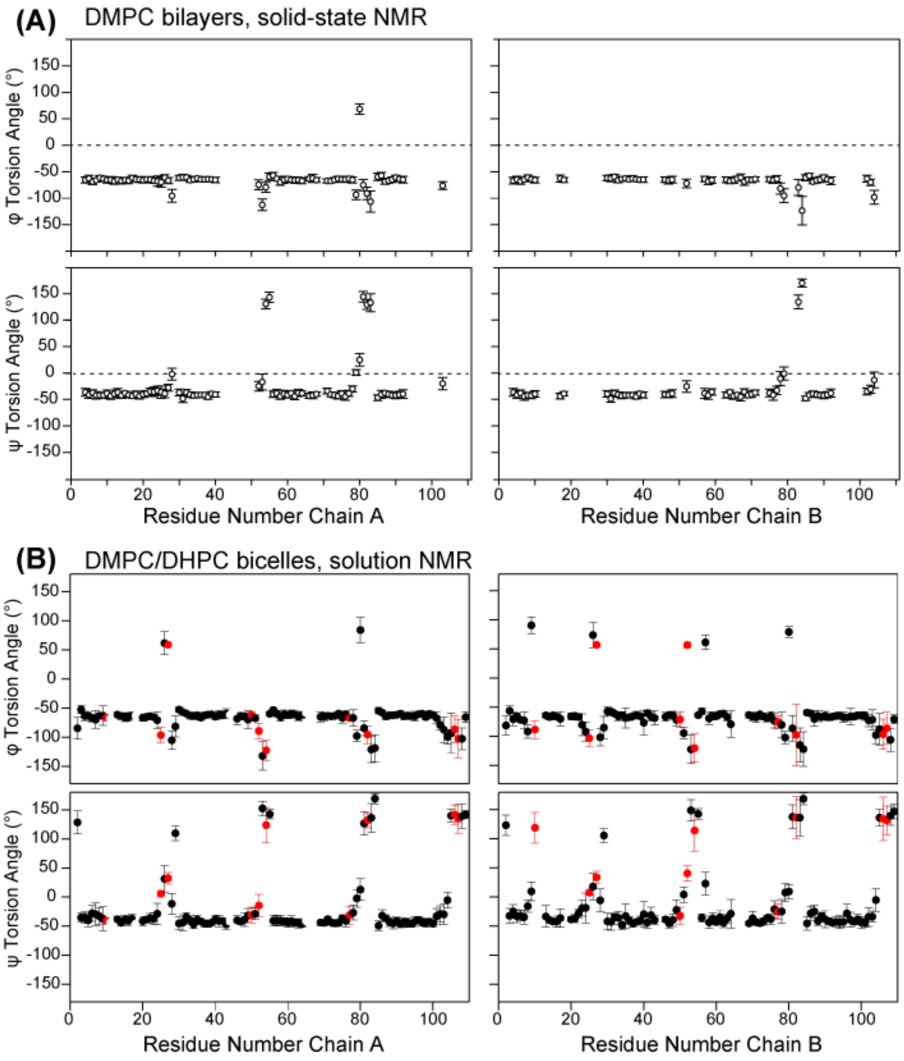

Supplementary Figure 7. Secondary structure of S64V-EmrE in lipid bilayers and bicelles are very similar. (**A**) TALOS (ϕ, ψ) torsion angles of DMPC-bound S64V-EmrE, obtained from Cα, CO, Cβ and ^{15}N chemical shifts measured from MAS NMR experiments at 285 K. Error bars represent the precision of the TALOS-N prediction, defined as one standard deviation for the (φ, ψ) angles among the best-matched peptides for each residue. (**B**) (ϕ, ψ) torsion angles of bicelle-bound S64V-EmrE, obtained from solution NMR experiments at 45˚C. The torsion angle values are colored black (good) and red (warn) based on TALOS-N classification. Error bars represent the precision of the TALOS-N prediction, defined as one standard deviation for the (φ, ψ) angles among the best-matched peptides for each residue.

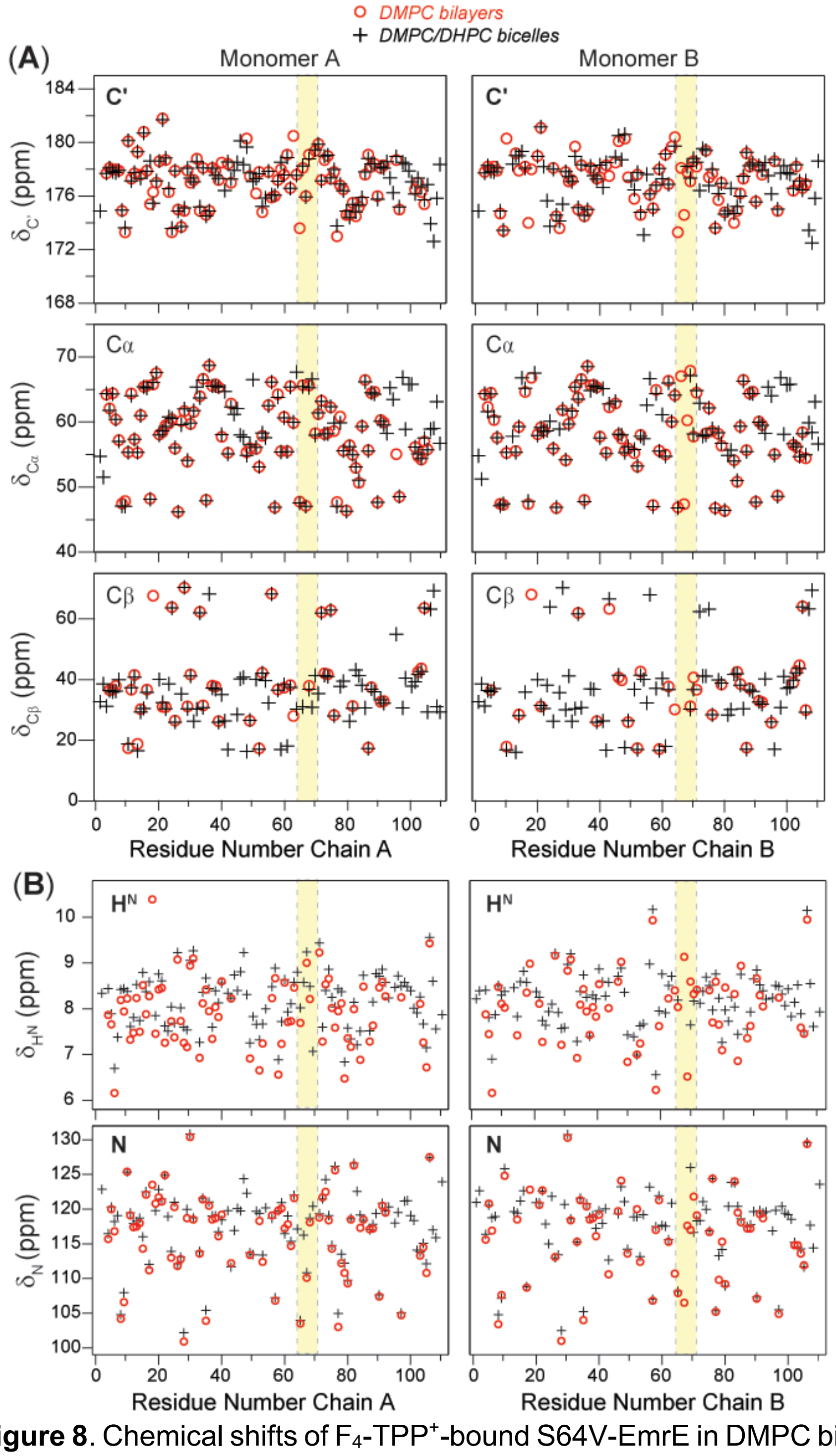

Supplementary Figure 8. Chemical shifts of F_4-TPP^+-bound S64V-EmrE in DMPC bilayers (red circles) versus DMPC/DHPC bicelles (black crosses), measured using MAS NMR and solution NMR experiments, respectively. (**A**) C', Cα and Cβ chemical shifts are very similar between the two environments. (**B**) H^N and ^{15}N chemical shifts, which are offset, as expected for the ~30°C temperature difference between the two sets of experiments. Monomer B (right) shows slightly larger N and H^N chemical shift changes for TM3 residues (shaded area) compared to TM3 residues in monomer A.

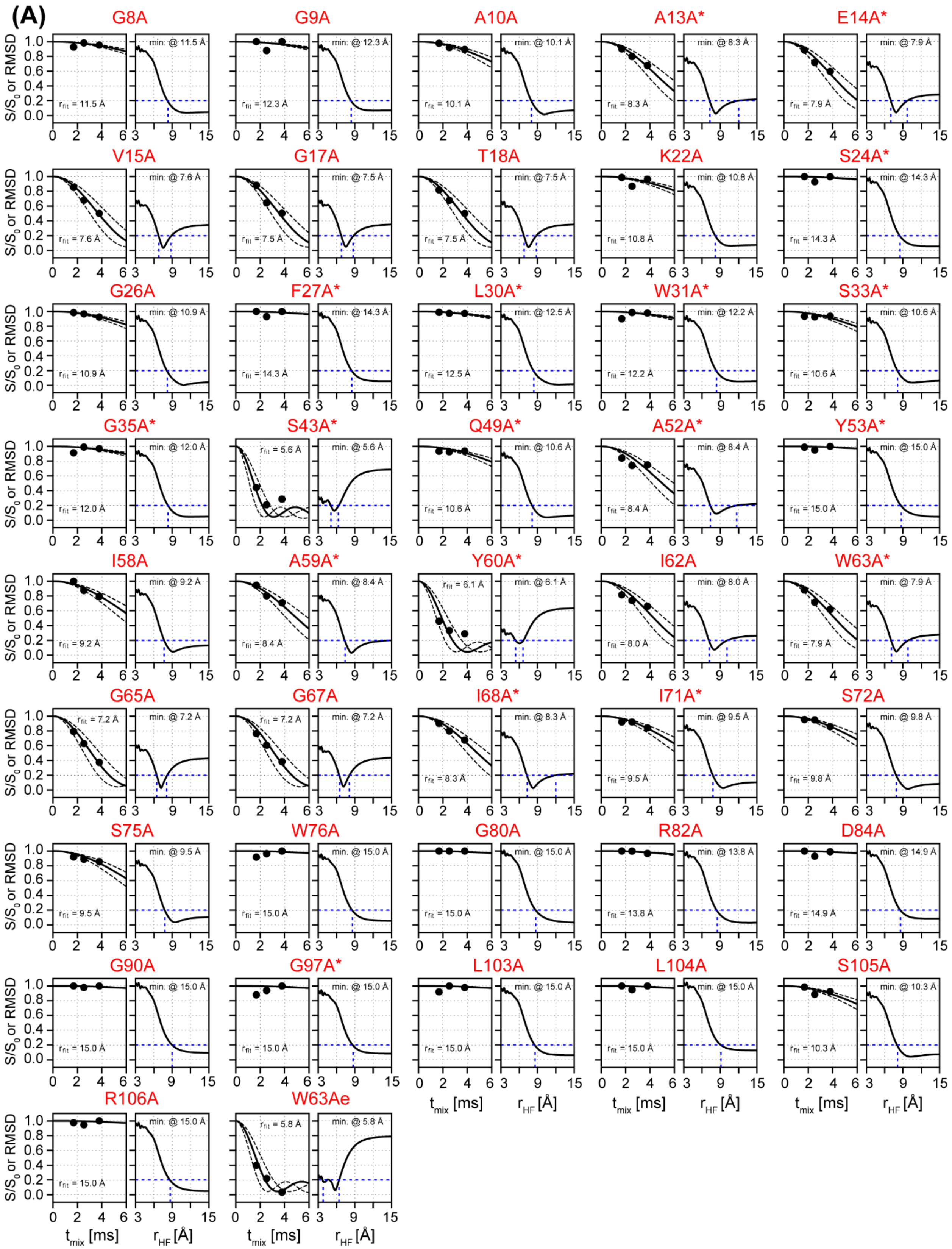
(A)
G8A
min. @ 11.5 Å
r_{fit} = 11.5 Å
G9A
min. @ 12.3 Å
r_{fit} = 12.3 Å
A10A
min. @ 10.1 Å
r_{fit} = 10.1 Å
A13A*
min. @ 8.3 Å
r_{fit} = 8.3 Å
E14A*
min. @ 7.9 Å
r_{fit} = 7.9 Å
V15A
min. @ 7.6 Å
r_{fit} = 7.6 Å
G17A
min. @ 7.5 Å
r_{fit} = 7.5 Å
T18A
min. @ 7.5 Å
r_{fit} = 7.5 Å
K22A
min. @ 10.8 Å
r_{fit} = 10.8 Å
S24A*
min. @ 14.3 Å
r_{fit} = 14.3 Å
G26A
min. @ 10.9 Å
r_{fit} = 10.9 Å
F27A*
min. @ 14.3 Å
r_{fit} = 14.3 Å
L30A*
min. @ 12.5 Å
r_{fit} = 12.5 Å
W31A*
min. @ 12.2 Å
r_{fit} = 12.2 Å
S33A*
min. @ 10.6 Å
r_{fit} = 10.6 Å
G35A*
min. @ 12.0 Å
r_{fit} = 12.0 Å
S43A*
r_{fit} = 5.6 Å
min. @ 5.6 Å
Q49A*
min. @ 10.6 Å
r_{fit} = 10.6 Å
A52A*
min. @ 8.4 Å
r_{fit} = 8.4 Å
Y53A*
min. @ 15.0 Å
r_{fit} = 15.0 Å
I58A
min. @ 9.2 Å
r_{fit} = 9.2 Å
A59A*
min. @ 8.4 Å
r_{fit} = 8.4 Å
Y60A*
r_{fit} = 6.1 Å
min. @ 6.1 Å
I62A
min. @ 8.0 Å
r_{fit} = 8.0 Å
W63A*
min. @ 7.9 Å
r_{fit} = 7.9 Å
G65A
r_{fit} = 7.2 Å
min. @ 7.2 Å
G67A
r_{fit} = 7.2 Å
min. @ 7.2 Å
I68A*
min. @ 8.3 Å
r_{fit} = 8.3 Å
I71A*
min. @ 9.5 Å
r_{fit} = 9.5 Å
S72A
min. @ 9.8 Å
r_{fit} = 9.8 Å
S75A
min. @ 9.5 Å
r_{fit} = 9.5 Å
W76A
min. @ 15.0 Å
r_{fit} = 15.0 Å
G80A
min. @ 15.0 Å
r_{fit} = 15.0 Å
R82A
min. @ 13.8 Å
r_{fit} = 13.8 Å
D84A
min. @ 14.9 Å
r_{fit} = 14.9 Å
G90A
min. @ 15.0 Å
r_{fit} = 15.0 Å
G97A*
min. @ 15.0 Å
r_{fit} = 15.0 Å
L103A
min. @ 15.0 Å
r_{fit} = 15.0 Å
L104A
min. @ 15.0 Å
r_{fit} = 15.0 Å
S105A
min. @ 10.3 Å
r_{fit} = 10.3 Å
R106A
min. @ 15.0 Å
r_{fit} = 15.0 Å
W63Ae
r_{fit} = 5.8 Å
min. @ 5.8 Å
S/S_0 or RMSD
t_{mix} [ms]
r_{HF} [Å]

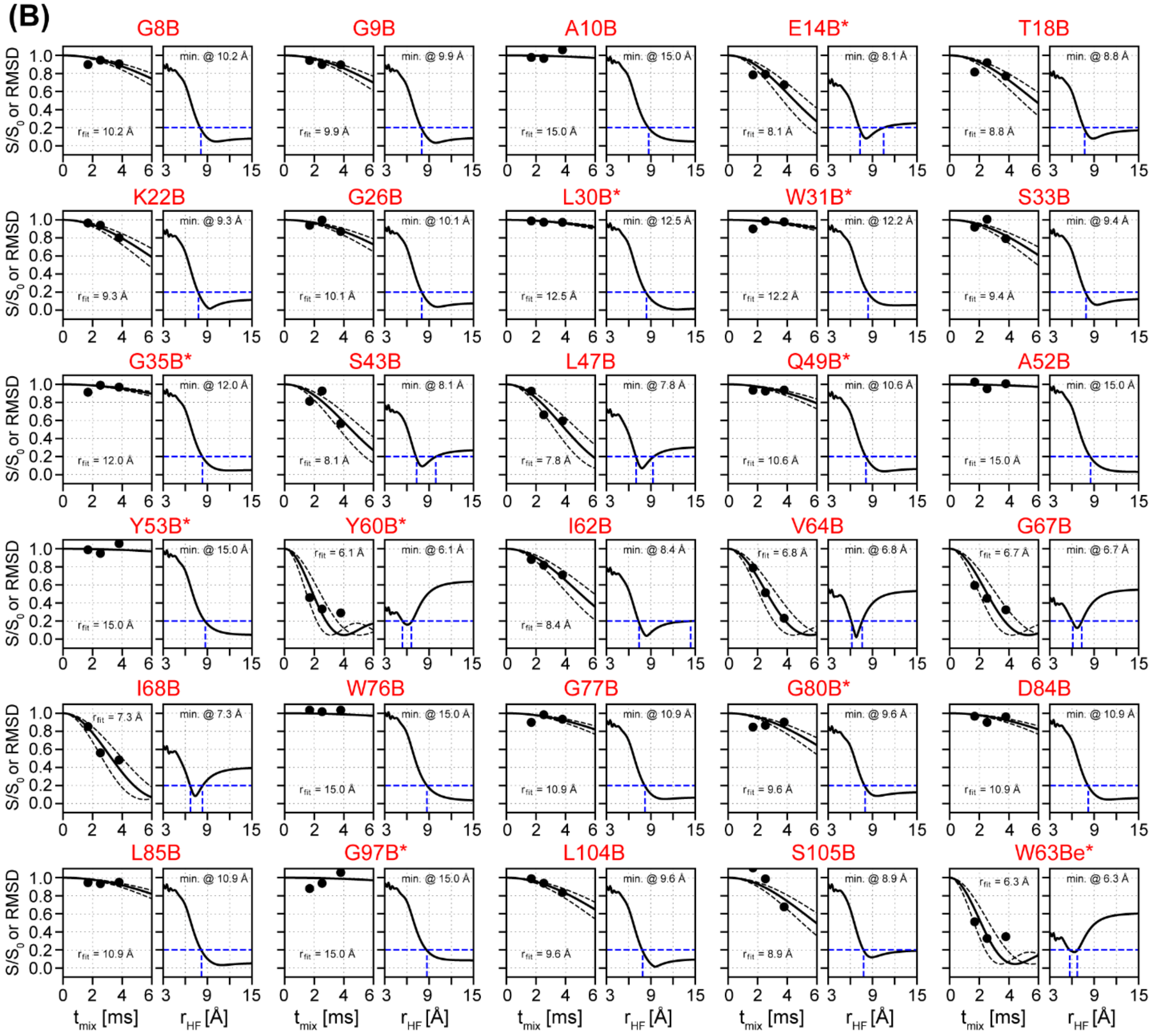

Supplementary Figure 9. Resolved H^N-F REDOR dephasing curves and RMSDs for extracting best-fit distances. (**A**) Monomer A data. (**B**) Monomer B data. For each resolved peak, the REDOR dephasing S/S_0 is plotted on the left and the RMSD between the measured and simulated intensities is shown on the right. Best-fit distance is extracted from the minimum RMSD position, and its simulated REDOR curve is overlaid with the experimental data on the left. Asterisks indicate residues whose signals are partially overlapped in the 2D spectra.

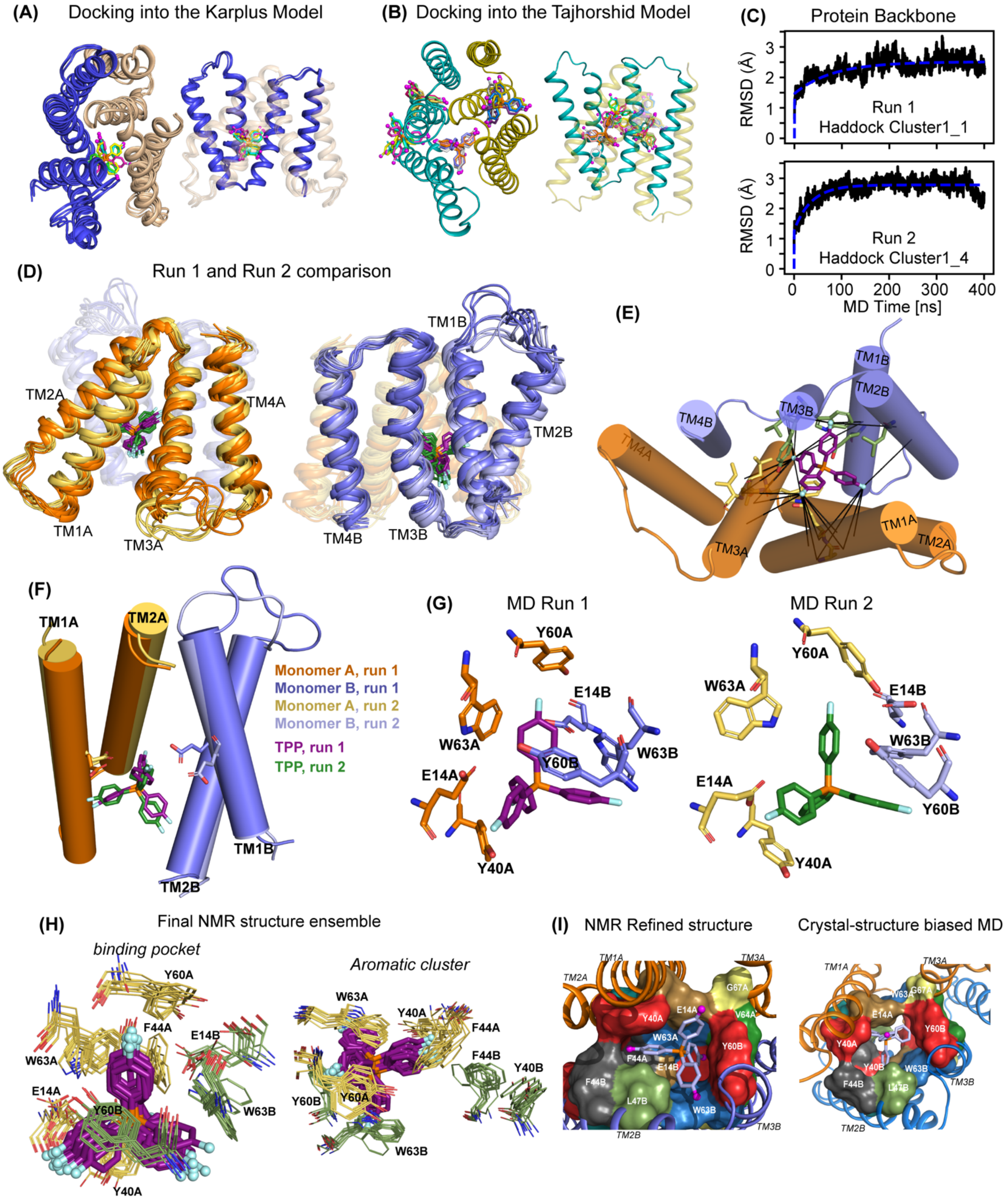

Supplementary Figure 10. Structure calculation of the EmrE-TPP$^+$ complex in lipid bilayers based on experimentally measured protein-substrates distances.

(A) HADDOCK docking of TPP$^+$ into the Karplus apo EmrE model [5]. TPP$^+$ location converges to a single site at the dimer interface in all 200 structures.

(B) HADDOCK docking of TPP^+ into the Tajkhorshid apo EmrE model [6]. Four clusters of TPP^+ locations are found, only one of which lies in the dimer interior.
(C) Protein backbone RMSD relative to the initial state as a function of simulation time during MD refinement. The overlaid biexponential fit shows that at 230 ns, the fitted value has reached 97.8% and 99.9% of the plateau value for run 1 and run 2, respectively. The structures are thus considered equilibrated between 230 and 400 ns.
(D) Comparison of equilibrated structure ensembles from MD simulation run 1 and run 2. States from run 1 are shown in darker colors ('slate', 'orange') with TPP^+ in purple, while states from run 2 are shown in lighter colors ('lightblue', 'yelloworange') with TPP^+ in green. Backbone differences between the two ensembles are mainly found in monomer B and are the largest in TM3 and TM2 helices.
(E) Measured protein-substrate distances up to 12 Å (black solid lines) at the drug-binding pocket. The lowest-violation NMR structure is shown.
(F) Overlay of TM1 and TM2 helices and F_4-TPP^+ between the two MD runs. The drug orientation is similar between the two runs and has a small RMSD of 0.61 Å for the TPP^+ center.
(G) Lowest-violation structure from each MD run, comparing the aromatic sidechain positions relative to F_4-TPP^+.
(H) Two views of the final NMR structure ensemble, oriented in the same fashion as Figure 5B. Key aromatic sidechains and F_4-TPP^+ are shown.
(I) Comparison of the TPP^+ binding site between the NMR structure model and the crystal-structure biased MD simulation model [5]. The binding site is viewed from the bottom of the dimer. Monomers A and B backbones are colored orange and blue, respectively. F_4-TPP^+ is shown in light blue, while the phenylene Hζ atoms are shown in pink.

References

1. Wu C, Wynne SA, Thomas NE, Uhlemann EM, Tate CG, Henzler-Wildman KA. Identification of an Alternating-Access Dynamics Mutant of EmrE with Impaired Transport. *J Mol Biol* **431**, 2777-2789 (2019).

2. Bazzone A, Barthmes M, Fendler K. SSM-Based Electrophysiology for Transporter Research. *Methods Enzymol* **594**, 31-83 (2017).

3. Robinson AE, Thomas NE, Morrison EA, Balthazor BM, Henzler-Wildman KA. New free-exchange model of EmrE transport. *Proc Natl Acad Sci U S A* **114**, E10083 (2017).

4. Thomas NE, Henzler-Wildman KA. Unlocking the Reversal Potential of Solid Supported Membrane Electrophysiology to Determine Transport Stoichiometry. *bioRxiv*, 2020.2005.2007.082438 (2020).

5. Ovchinnikov V, Stone TA, Deber CM, Karplus M. Structure of the EmrE multidrug transporter and its use for inhibitor peptide design. *Proc Natl Acad Sci U S A* **115**, E7932 (2018).

6. Vermaas JV, Rempe SB, Tajkhorshid E. Electrostatic lock in the transport cycle of the multidrug resistance transporter EmrE. *Proc Natl Acad Sci U S A* **115**, E7502 (2018).
