# Supplementary material for: Structure and dynamics of the drug-bound bacterial transporter EmrE in lipid bilayers
Source: Nat Commun. 2021 Jan 8;12:172. doi: 10.1038/s41467-020-20468-7 (PMC7794478; doi:10.1038/s41467-020-20468-7)
Supplement: Supplementary file 3 — Description of additional supplementary files [file 41467_2020_20468_MOESM3_ESM.docx]

**Description of Additional Supplementary files**

**Title: Dataset 1**.

Description: HN, 15N and 13 C Chemical shifts (ppm) of DMPCbound S64V EmrE with F4-TPP+. The 1H and 13C chemical shifts are reported on the DSS scale whereas the 15N chemical shifts are reported on the liquid ammonia scale.

**Title: Dataset 2.**

Description: Distance constraints used in HADDOCK calculations and the resulting structure-based HF pair assignment. rHF is the best-fit experimental REDOR distance, while δLL and δUP are the uncertainties for the lower and upper limits, respectively.

**Title:**  **Dataset 3.**

Description: Distance constraints used in GROMACS simulations of F4-TPP+ bound EmrE structure. Distances r0 and r1 define the lower and upper limits between which no energy penalty is applied.

**Title: Dataset 4.**

Description: Detailed parameters for the solid-state NMR experiments for resonance assignment and distance measurements of F4- TPP+ bound EmrE in DMPC bilayers.
