# Supplementary material for: Structure and dynamics of the drug-bound bacterial transporter EmrE in lipid bilayers
Source: Nat Commun. 2021 Jan 8;12:172. doi: 10.1038/s41467-020-20468-7 (PMC7794478; doi:10.1038/s41467-020-20468-7)
Supplement: Supplementary file 4 — Reporting Summary [file 41467_2020_20468_MOESM4_ESM.pdf]

nature research

Corresponding author(s): Mei Hong and Katherine A. Henzler-Wildman

Last updated by author(s): 11/29/2020

Reporting Summary

Nature Research wishes to improve the reproducibility of the work that we publish. This form provides structure for consistency and transparency in reporting. For further information on Nature Research policies, see our Editorial Policies and the Editorial Policy Checklist.

Statistics

For all statistical analyses, confirm that the following items are present in the figure legend, table legend, main text, or Methods section.

n/a	Confirmed	
☐	☒	The exact sample size (n) for each experimental group/condition, given as a discrete number and unit of measurement
☐	☒	A statement on whether measurements were taken from distinct samples or whether the same sample was measured repeatedly
☒	☐	The statistical test(s) used AND whether they are one- or two-sided<br>*Only common tests should be described solely by name; describe more complex techniques in the Methods section.*
☒	☐	A description of all covariates tested
☒	☐	A description of any assumptions or corrections, such as tests of normality and adjustment for multiple comparisons
☐	☒	A full description of the statistical parameters including central tendency (e.g. means) or other basic estimates (e.g. regression coefficient) AND variation (e.g. standard deviation) or associated estimates of uncertainty (e.g. confidence intervals)
☒	☐	For null hypothesis testing, the test statistic (e.g. F, t, r) with confidence intervals, effect sizes, degrees of freedom and P value noted<br>*Give P values as exact values whenever suitable.*
☐	☐	For Bayesian analysis, information on the choice of priors and Markov chain Monte Carlo settings
☒	☐	For hierarchical and complex designs, identification of the appropriate level for tests and full reporting of outcomes
☒	☐	Estimates of effect sizes (e.g. Cohen's d, Pearson's r), indicating how they were calculated

Our web collection on statistics for biologists contains articles on many of the points above.

Software and code

Policy information about availability of computer code

Data collection	NMR data acquisition: Topspin 3.2 and 3.6
Data analysis	NMR data processing and plotting: Topspin 2.1, 3.2 and 3.6<br>NMR peak picking and resonance assignment: Sparky 3<br>Torsion angle predictions: TALOS-N webserver<br>1H-19F REDOR simulations: SIMPSON 4.1<br>Converting experimental restraints to GROMACS input files: custom code written with Python 3.6<br>Docking: HADDOCK 2.4<br>MD preparation: CHARMM-GUI 3.1<br>Structure calculation: GROMACS 2019.1<br>Pymol 2.3.5

For manuscripts utilizing custom algorithms or software that are central to the research but not yet described in published literature, software must be made available to editors and reviewers. We strongly encourage code deposition in a community repository (e.g. GitHub). See the Nature Research guidelines for submitting code & software for further information.

Data

Policy information about availability of data

All manuscripts must include a data availability statement. This statement should provide the following information, where applicable:

- Accession codes, unique identifiers, or web links for publicly available datasets
- A list of figures that have associated raw data
- A description of any restrictions on data availability

NMR chemical shifts and the resulting torsion angles, distance restraints for EmrE have been deposited in the Biological Magnetic Resonance Bank (BMRB) with ID number 50411. The structural coordinates for substrate-bound EmrE has been deposited in the Protein Data Bank with accession codes 7JK8.

Field-specific reporting

Please select the one below that is the best fit for your research. If you are not sure, read the appropriate sections before making your selection.

☒ Life sciences ☐ Behavioural & social sciences ☐ Ecological, evolutionary & environmental sciences

For a reference copy of the document with all sections, see nature.com/documents/nr-reporting-summary-flat.pdf

Life sciences study design

All studies must disclose on these points even when the disclosure is negative.

Sample size	NMR samples contained > 10^16 molecules. The NMR spectra report the average properties of all molecules in the sample. A large number of scans were obtained for each NMR spectrum to generate signal-to-noise ratios of at least 5 to 1. Therefore all reported spectra are inherently reproducible. All protein samples with their isotopic labeling pattern and protein/lipid ratios are reported in Table S4. The number of scans used to average each spectrum are given in Table S4.
Data exclusions	No data was excluded.
Replication	The protein samples and NMR spectra were highly replicable. Three protein samples with different isotopic labeling schemes and protein to lipid ratios were used for this study. They give the same fingerprint 2D NMR spectra over the course of 1.5 years. No chemical shift changes or sensitivity changes were observed during this time.
Randomization	Not applicable to this NMR study
Blinding	Not applicable to this NMR study.

Reporting for specific materials, systems and methods

We require information from authors about some types of materials, experimental systems and methods used in many studies. Here, indicate whether each material, system or method listed is relevant to your study. If you are not sure if a list item applies to your research, read the appropriate section before selecting a response.

Materials & experimental systems

n/a	Involved in the study
☒	☐ Antibodies
☒	☐ Eukaryotic cell lines
☒	☐ Palaeontology and archaeology
☒	☐ Animals and other organisms
☒	☐ Human research participants
☒	☐ Clinical data
☒	☐ Dual use research of concern

Methods

n/a	Involved in the study
☒	☐ ChIP-seq
☒	☐ Flow cytometry
☒	☐ MRI-based neuroimaging
